# Inflammatory Cytokines and BDNF Response to High-Intensity Intermittent Exercise: Effect the Exercise Volume

**DOI:** 10.3389/fphys.2016.00509

**Published:** 2016-11-04

**Authors:** Carolina Cabral-Santos, Carlos I. M. Castrillón, Rodolfo A. T. Miranda, Paula A. Monteiro, Daniela S. Inoue, Eduardo Z. Campos, Peter Hofmann, Fábio S. Lira

**Affiliations:** ^1^Exercise and Immunometabolism Research Group, Departamento de Educação Física, Universidade Estadual Paulista – Presidente PrudenteSão Paulo, Brasil; ^2^Laboratório de Fisioterapia Desportiva, Faculdade de Ciências e Tecnologia, Departamento de Fisioterapia, Universidade Estadual Paulista – Presidente PrudenteSão Paulo, Brasil; ^3^Department of Physical Education, Federal University of PernambucoRecife, Brazil; ^4^Exercise Physiology, Training and Training Therapy Research Group, Institute of Sport Science, University of GrazGraz, Austria

**Keywords:** high-intensity intermittent exercise, inflammatory response, metabolic response, cytokines, low-grade inflammation

## Abstract

The purpose of this study was to compare the effects of two similar high-intensity intermittent exercises (HIIE) but different volume 1.25 km (HIIE1.25) and 2.5 km (HIIE2.5) on inflammatory and BDNF responses. Ten physically active male subjects (age 25.22 ± 1.74 years, body mass 78.98 ± 7.31 kg, height 1.78 ± 0.06 m, VO_2peak_ 59.94 ± 9.38 ml·kg·min^−1^) performed an incremental treadmill exercise test and randomly completed two sessions of HIIE on a treadmill (1:1 min at vVO_2max_ with passive recovery). Blood samples were collected at rest, immediately and 60-min after the exercise sessions. Serum was analyzed for glucose, lactate, IL-6, IL-10, and BDNF levels. Blood lactate concentrations was higher immediately post-exercise compared to rest (HIIE1.25: 1.69 ± 0.26–7.78 ± 2.09 mmol·L^−1^, and HIIE2.5: 1.89 ± 0.26–7.38 ± 2.57 mmol·L^−1^, *p* < 0.0001). Glucose concentrations did not present changes under the different conditions, however, levels were higher 60-min post-exercise than at rest only in the HIIE1.25 condition (rest: 76.80 ± 11.14–97.84 ± 24.87 mg·dL^−1^, *p* < 0.05). BDNF level increased immediately after exercise in both protocols (HIIE1.25: 9.71 ± 306–17.86 ± 8.59 ng.mL^−1^, and HIIE2.5: 11.83 ± 5.82–22.84 ± 10.30 ng.mL^−1^). Although both exercises increased IL-6, level percent between rest and immediately after exercise was higher in the HIIE2.5 than HIIE1.25 (30 and 10%; *p* = 0.014, respectively). Moreover, IL-10 levels percent increase between immediately and 60-min post-exercise was higher in HIIE2.5 than HIIE1.25 (37 and 10%; *p* = 0.012, respectively). In conclusion, both HIIE protocols with the same intensity were effective to increase BDNF and IL-6 levels immediately after exercise while only IL-10 response was related to the durantion of exercise indicanting the importance of this exercise prescription variable.

## Introduction

Classically, endurance training programs in non-athletes are aiming to promote health and prevent life style related diseases (Pedersen and Saltin, 2015). Such training programs need much time, but this does not necessarily mean that spending hours performing exercise will intensify the gains, when in fact the opposite may happen. As known for resistance exercise working until clear fatigue gives higher adaptation compared to sub-maximal number of repetitions. Similarly, Lyakh et al. (2015) discusses that a significant decrease in total training volume allows to focus on qualitative aspects of a training process.

A significantly reduced training volume increases the ability to recover more quickly, although the stimulus for the muscles may be even highly intense. HIIE is characterized by repeated bouts of short-duration (30–60 s) exercises performed at high to severe intensity (i.e., ≥90% of VO_2max_) interspersed by short periods of passive or active recovery (Zwetsloot et al., 2014). This method is considered a time-efficient way to improve health outcomes. Furthermore, Cockcroft et al. (2015) showed that a single bout of HIIE (8 × 1-min at 90% of the peak power, interspersed by 1.25 min recovery at 20 W) is an effective alternative to moderate intensity exercise to improve glucose tolerance, insulin sensitivity, and fat oxidation immediately after exercise. Studies have shown that high intensity intermittent exercise (HIIE) alone or combined with strength exercise promoted changes in metabolic and inflammatory responses (Meckel et al., 2009, 2011; Zwetsloot et al., 2014; Cabral-Santos et al., 2015; Lira et al., 2015; Wadley et al., 2016). To induce specific acute physiological responses an optimal ratio of intensity and duration of high intensity intervals and recovery as well as the resulting mean workload have to be optimized aiming for specific training adaptations (Tschakert and Hofmann, 2013).

Brain derived neurotrophic factor (BDNF), a neurotrophin family member who is involved in neuroprotection and neurogenesis, has several actions on cell functions, including energy metabolism, promoting glucose uptake via mitochondrial biogenesis, contributing to cellular homeostasis, particularly in the central nervous system (CNS; Marosi and Mattson, 2014). Saucedo Marquez et al. (2015) demonstrated that only one HIIE session was able to increase BDNF levels immediately after exercise performing a protocol with intervals of 1 min at 90% of maximal work load, altering with 1 min active rest (low load) during 20 min but a duration effect was not investigated.

Additionally, Monocyte Chemo-attractant Protein-1 (MCP-1) is related to inflammatory mediators and is a necessary component of the inflammatory response required for adipose tissue protection (Cranford et al., 2015). Combining exercise and dietary intake restriction is likely to prevent an influx of macrophages by reducing the number of fat cells (Ko and Kim, 2013; Ahn and Kim, 2014). Maharaj et al. (2016) observed a significant elevation across time in plasma MCP-1 and IL-6 after aerobic exercise (30-min bout performed at 75%VO_2max_), suggesting that exercise could mediate an innate immune response related to monocyte recruitment and inflammatory mediators.

Besides health benefits, acute exercise enhances interleukin 6 (IL-6) concentration by increasing its production in skeletal muscle, that acts in the regulation of muscle energetic status (Pedersen and Febbraio, 2009). Additionally, IL-6 promotes enhancements in anti-inflammatory cytokines, such as interleukin 10 (IL-10), related to prevent the exacerbation of the pro-inflammatory milieu, blocking a possible persistent inflammatory status, and reduces tumor necrosis factor alpha (TNF-α; Pedersen and Febbraio, 2009; Rosa Neto et al., 2011; Lira et al., 2012). Recently we compared the inflammatory response of HIIE (1:1 at 100% at sVO_2peak_) and moderate-intensity continuous exercise (70% of sVO_2peak_) with matched volume (5 km), and showed that both exercise protocols promoted an anti-inflammatory response, by augmenting IL-10 and the IL-10/TNF-α ratio (Cabral-Santos et al., 2015). This effects can be utilized as strategies for different populations, such as in patients suffering from obesity, diabetes, or dyslipidemia. From a practical point of view it is interesting to establish the optimal duration of such a HIIE to be sufficient to augment an anti-inflammatory response. Thus, the present study aimed to determine whether the same exercise intensity and mode (HIIE) but different duration and degree of fatigue (1.25- and 2.5-km) may induce similar anti-inflammatory (IL-6, IL-10, MCP-1) and metabolic (lactate and glycemic) responses in young men.

## Materials and methods

### Participants

Ten physically active male subjects volunteered for the present study. They presented a health and neuromuscular status that ensured their ability to complete the study protocol. All procedures performed in the study were approved by the Research Ethics Committee for studies involving human participants of the Universidade Estadual Paulista (UNESP)—campus Presidente Prudente/SP, and were in accordance with the ethical standards of World Medical Association Declaration of Helsinki. Written informed consent was obtained from all subjects after they had been informed about the purpose and risks of the study.

Before conducting the study we checked the sample size needed (*n* = 10) using the G^*^Power 3.1 software (Düsseldorf, Germany) to guarantee an 80% power and a 5% significance level based on a study that measured the IL-6 pre and immediately post exercise using a similar protocol from a previous study (Cabral-Santos et al., 2015).

### Bioelectrical impedance

Bioelectrical Impendence in individuals was measured using the octopolar InBody 720 Composition Analyzer (Copyright®, 1996–2006, by Biospace Corporation, USA). The participant's age, sex, and height were entered into the machine. The participants stood barefoot on the metal footplate and held the handles with their arms relaxed by their sides. Once impedance was measured, the results of fat mass and %body fat was printed. All anthropometric measurements were checked by the same person throughout the study to minimize interpersonal variations. Participants were asked to abstain from eating or drinking for at least 2 h as well as to refrain from moderate or vigorous exercise for 24 h before all testing. They were told to obtain a restful night's sleep, remain well-hydrated, refrain from alcohol, and eat a regular meal in the morning before testing, all according to the manufacturer's recommendations.

### Maximal endurance running test

The subjects performed an incremental test to volitional fatigue (Panissa et al., 2013). The initial treadmill (Inbramed, modelo MASTER CI, Brazil) speed was set at 8.0 km·h-1^−1^ with 1% inclination, and it was increased by 1 km·h-1^−1^ after each 2-min until the participant could no longer continue. Strong verbal encouragement was given during the test. The oxygen uptake was measured (Quark PFT, Cosmed, Rome, Italy) throughout the test and the average of the last 30 s was defined as peak oxygen uptake (VO_2peak_). The speed associated with VO_2peak_ (sVO_2peak_) was assumed as the speed at the last stage completed or when the subject was not able to finish the 2-min stage, the speed was expressed according to the permanence time in the last stage, determined as the following: sVO_2peak_ = speed of last stage complete + [(time (seconds) performed in the last stage/120) ^*^ 1] (Kuipers et al., 1985). Heart rate was also continuously recorded throughout the tests (Polar Vantage NV, Electro Oy, Finland). The 6–20 Borg scale (Borg, 1982) was used to obtain the rating of perceived exertion during the test.

### High-intensity intermittent exercise

After the incremental test, the subjects completed two experimental protocols, applied in randomized cross-over order sessions, separated by at least 72 h. The protocol was performed intermittently with subjects running on a treadmill for 1 min at 100% at sVO_2peak_, interspersed by 1 min of passive recovery (which subjects jumped to the side of the treadmill by holding onto the bars for the recovery minute and remained standing or seated). They ran until they had completed the 1.25 or 2.5 km distance. For both exercise trials, the subjects performed a warm-up consisting of running at 50% of sVO_2peak_ for 5 min, at 1% inclination, and after that thefirst- 1-min interval exercise bout was started.

Due to the influence of time of day, all tests took place at the same time of the day (between 10:00 a.m. and 12:00 p.m.), at an average temperature between 20 and 24°C. The subjects were instructed to abstain from strenuous exercise for at least 24 h prior to each exercise session, and were encouraged to maintain their usual nutritional and hydration routines. Moreover, they were also request not to ingest stimulants (tea, coffee, soda, chocolate, chocolate powder), or alcoholic beverages during this period.

### Dietary intake assessment

Diet was not standardized. However, participants were required to eat 3 h prior to all testing sessions. Participants were instructed by a nutritionist how to complete the food records and were required to record all foods consumed in last night and on the day of each testing session. Nutrition data was analyzed for energy intake and macronutrient distribution using the NutWin software version 1.5 (Programa de Apoio à Nutrição, Universidade Federal de São Paulo, Brazil, 2002).

### Blood sampling and analyses

The blood samples were collected by a trained nurse at rest, immediately and 60 min after acute exercise sessions during HIIE1.25 and HIIE2.5. The blood samples (15 ml) were immediately allocated into two 5 mL vacutainer tubes (Becton Dickinson, BD, Juiz de Fora, MG, Brazil) containing EDTA for plasma separation and one 5 mL dry vacutainer tube for serum separation. The tubes were centrifuged at 3.500 rpm for 15 min at 4°C, and plasma and serum samples were stored at −20°C until analysis. Cytokines IL-6, IL-10, BDNF, and MCP-1 were assessed using ELISA commercial kits (R&D Systems, 614 McKinley Place NE, Minneapolis, MN 55413, USA). Glucose and lactate were assessed using commercial kits (Labtest®, São Paulo, Brazil).

### Statistical analysis

Data normality were verified using the Shapiro-Wilk test and descriptive data are shown as means and standard deviation. The independent *t*-test was used to compare the differences in metabolic variables at baseline between the two protocols.

One-way analysis of variance (ANOVA), with Tukey's *post-hoc* analysis were used to examine differences in metabolic variables at different moments (at rest, immediately after and 60 min after exercise) of each HIIE1.25 and HIIE2.5 effort. The same analysis was used to compare the differences (Δ) in metabolic variables between the HIIE1.25 and HIIE2.5. The repeated measures analysis was used to compare the interaction 2 (HIIT: 1.25, 2.5) × 3 (time: pre, post, 60 post). Statistical significance was set at 5% for all the analyses and the calculations were conducted using SPSS, version 17.0 (SPSS Inc., Chicago, IL).

## Results

Table 1 presents the subjects characteristics and mean values of both protocols of this study.

**Table 1:** **Subjects characteristics (***n*** = 10)**.

**Variable**	**Mean ±*SD***
Age (years)	25.22±1.74
Body Mass (kg)	78.98±7.31
Height (m)	1.78±0.06
BMI (kg·m^2^)	24.85±2.00
Fat Mass (%)	17.93±4.69
VO_2_peak (ml·kg·min^−1^)	59.94±9.38
sVO_2_peak (km·h^−1^)	13.61±1.06
Duration session HIIE_1.25_ (min)	10.08±0.81
Duration session HIIE_2.5_(min)	21.15±1.62
Bouts per session HIIE_1.25_	5.54±0.40
Bouts per session HIIE_2.5_	11.08±0.81
Heart rate rest (bpm)	65.40±7.26
Heart rate final HIIE_1.25_(bpm)	170.0±6.94
Heart rate final HIIE_2.5_(bpm)	175.7±9.09

*BMI, Body Mass Index; VO_2_max, maximal oxygen uptake; sVO_2max_, speed associated with maximal oxygen uptake; bpm, beat per minutes*.

The total food intake from last dinner and breakfast (at least 3 h before the test), expressed in kcal, were not significantly different between both conditions (HIIE1.25: 980 ± 288 kcal vs. HIIE2.5: 1048 ± 198 kcal; *p* = 0.731) and the macronutrient distribution such as carbohydrates (HIIE1.25 = 124 ± 54 vs. HIIE2.5 = 101 ± 51 grams, *p* = 0.417), protein (HIIE1.25 = 54 ± 35 vs. HIIE2.5 = 60 ± 22 grams, *p* = 0.813), and lipids (HIIE1.25 = 29 ± 13 vs. HIIE2.5 = 41 ± 16 grams, *p* = 0.190).

**Figure 1:**
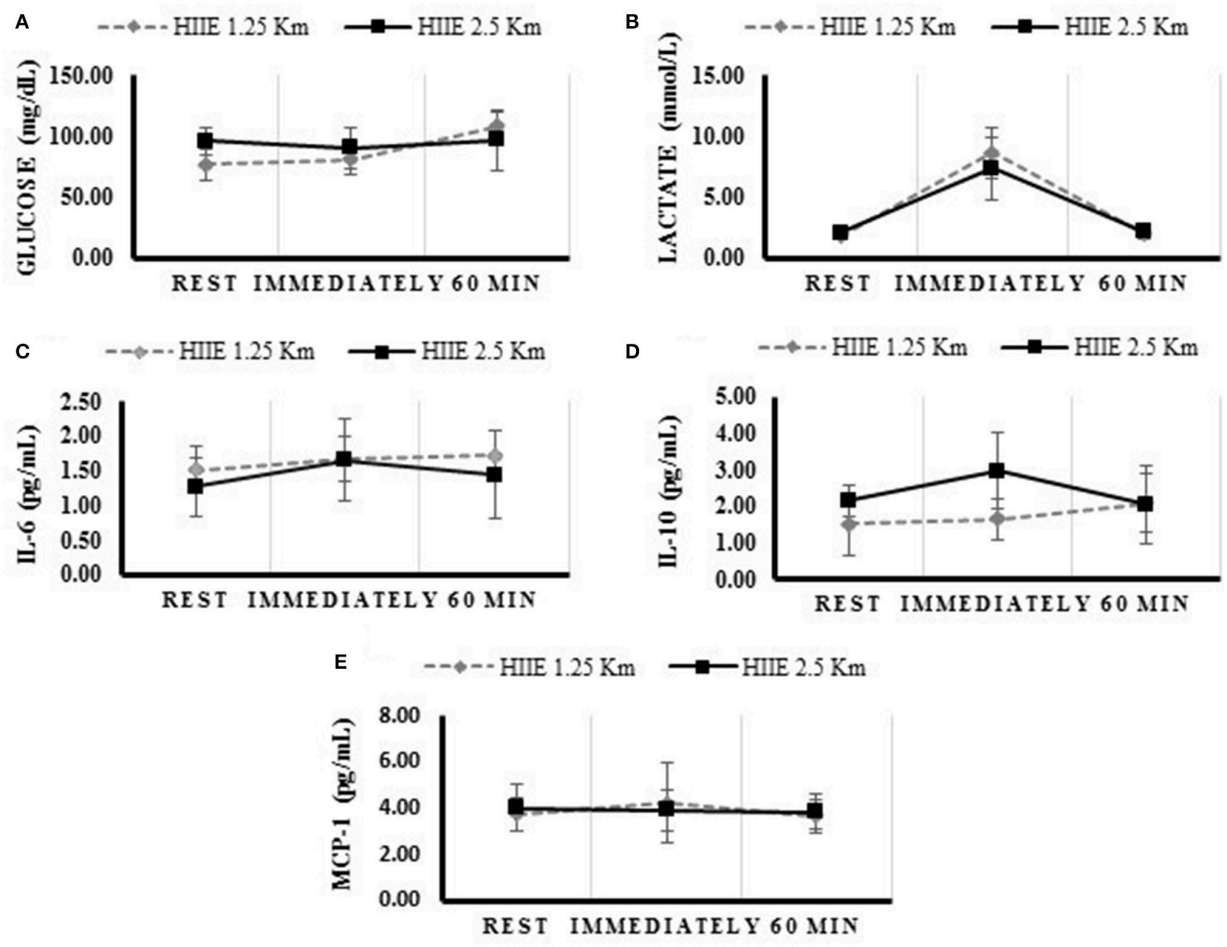
**Concentrations of metabolic and inflammatory profile during rest, immediately and 60 min after the HIIE 1.25 and HIIE 2.5 km**. Glucose **(A)**, Lactate **(B)**, Interleukine 6 **(C)**, Interleukine 10 **(D)**, Monocyte Chemo-attractant Protein 1 **(E)**.

The concentration of glucose, lactate, IL-6, IL-10, BDNF, and MCP-1 at rest, immediately, and 60 min after the exercise, are presented in Table 2 and Figure 1. The variables analyzed were similar at baseline. Both HIIE exercises increased lactate and IL-6 concentration immediately after the exercise, while only the HIIE2.5 elevated IL-10 (*p* = 0.023). Compared with at rest, glucose remained elevated 60 min after the HIIE1.25 (*p* = 0.007), and IL-6 after the HIIE2.5 (*p* = 0.019).

**Table 2:** **Alterations of metabolic variables after HIIE 1.25 and 2.5 km (***n*** = 10)**.

**1.25 km**	**Rest mean (*SD*)**	**Immediately mean (*SD*)**	**60 min mean (*SD*)**
Glucose (mg/dL)	76.80 (11.14)	72.77 (17.32)	97.84 (24.87)^a^^,^ ^b^
Lactate (mmol/L)	1.69 (0.26)	7.78 (2.09)^a^	1.79 (0.42)^b^
IL-6 (pg/mL)	1.51 (0.34)	1.67 (0.31)^a^	1.72 (0.34)
IL-10 (pg/mL)	1.51 (0.86)	1.67 (0.56)	2.09 (0.80)
MCP-1 (pg/mL)	4.81 (0.72)	5.46 (1.76)^a^	4.74 (0.72)^b^
BDNF (ng/mL)	9.71 (3.06)	17.86 (8.59)^a^	8.82 (2.74)^b^
**2.5 km**	**Rest mean (*SD*)**	**Immediately mean (*SD*)**	**60 min mean (*SD*)**
Glucose (mg/dL)	86.92 (12.77)	81.39 (12.67)	87.45 (11.29)
Lactate (mmol/L)	1.89 (0.26)	7.38 (2.57)^a^	1.97 (0.49)^b^
IL-6 (pg/mL)	1.55 (0.42)	2.03 (0.59)^a^	1.79 (0.62)
IL-10 (pg/mL)	2.16 (0.43)	2.96 (1.04)^a^	2.04 (1.04)^b^
MCP-1 (pg/mL)	5.14 (1.01)	5.05 (0.87)	4.95 (0.75)
BDNF (ng/mL)	11.83 (5.82)	22.84 (10.30)^a^	8.40 (3.04)^b^

a *Tukey's post-hoc test with p < 0.05 compared to rest*;

b *Tukey's post-hoc test with p < 0.05 compared between Immediately with 60 min*.

Table 3 shows the differences between the HIIE1.25 and HIIE2.5 for glucose, IL-10, IL-6, lactate, BDNF, and MCP-1, as well as, differences between time points (rest, immediately and 60 min) for glucose, IL-10, IL-6, lactate, BDNF, and MCP-1 too. The variables IL-6 and IL-10, showed interaction between the group (HIIE1.25 vs. 2.5) and the time (rest, immediately and 60 min).

**Table 3:** **Difference between the deltas of the metabolic variables of HIIE 1.25 and 2.5 km (***n*** = 10)**.

		**HIIE 1.25 km Δ mean (*SD*)**	**HIIE 2.5 km Δ mean (*SD*)**	**Effect**	***F***	***P***	**Effect size**
Glucose (mg/dL)	Rest and immediately	−3.27 (13.62)	−5.54 (18.30)	**Group**	**8.450**	**0.003**	**0.514**
	Rest and 60 min	21.78 (18.13)	0.54 (10.42)	**Time**	**8.241**	**0.011**	**0.326**
	Immediately and 60 min	25.05 (22.19)	6.07 (11.30)	Group × Time	1.444	0.246	0.078
Lactate (mmol/L)	Rest and Immediately	6.09 (2.05)	4.75 (3.38)	**Group**	**45.396**	<**0.001**	**0.842**
	Rest and 60 min	0.11 (0.50)	0.08 (0.43)	**Time**	**80.498**	<**0.001**	**0.817**
	Immediately and 60 min	−5.98 (1.77)	−4.67 (3.17)	Group × Time	1.234	0.281	0.064
IL-6 (pg/mL)	Rest and Immediately	0.15 (0.14)	0.47 (0.31)	**Group**	**14.495**	<**0.001**	**0.659**
	Rest and 60 min	0.21 (0.39)	0.25 (0.25)	**Time**	**12.384**	**0.003**	**0.436**
	Immediately and 60 min	0.05 (0.35)	−0.23 (0.32)	**Group ×Time**	**6.764**	**0.019**	**0.297**
IL-10 (pg/mL)	Rest and Immediately	0.15 (0.42)	0.80 (0.85)	**Group**	**4.226**	**0.035**	**0.360**
	Rest and 60 min	0.58 (0.96)	−0.11 (0.98)	**Time**	**4.649**	**0.047**	**0.225**
	Immediately and 60 min	0.42 (0.78)	−0.91 (1.19)	**Group ×Time**	**8.904**	**0.009**	**0.358**
MCP-1 (pg/mL)	Rest and Immediately	0.65 (1.69)	−0.10 (0.41)	Group	1.559	0.253	0.221
	Rest and 60 min	−0.06 (0.16)	−0.19 (0.43)	Time	1.104	0.314	0.084
	Immediately and 60 min	−0.71 (1.70)	−0.09 (0.23)	Group × Time	1.113	0.312	0.085
BDNF (ng/mL)	Rest and Immediately	9.02 (7.05)	11.01 (4.99)	**Group**	**24.634**	<**0.001**	**0.755**
	Rest and 60 min	−1.08 (2.60)	−4.27 (6.01)	**Time**	**45.097**	<**0.001**	**0.726**
	Immediately and 60 min	−10.11 (8.32)	−15.2 (9.75)	Group × Time	1.116	0.305	0.062

*Bold values = significant difference with p < 0.05*.

## Discussion

Recently, a study by Wadley et al. (2016) suggest that the magnitude of increase in cytokines in response to exercise is dependent on exercise intensity. In our study we questioned whether there is a threshold like level of HIIE intensity vs. time combination required to observe such effect. The main finding of the present study suggests that the increase in plasma concentrations of IL-10 occurs in a duration-dependent manner, as that same intensity in the task but twice the duration gave differences in the response profile. HIIE2.5 maintained temporal differences that induced higher IL-10 levels (indicated by the highest peak in the phase immediately following exercise) when compared to HIIE1.25, which only presented a trend for an increase. However, after 60-min, the IL-10 levels had returned to baseline values in both protocols.

High-intensity intermittent training has been shown to be effective for improving body composition parameters, physical fitness, and lipid profile (Martins et al., 2016). Acute studies have proposed that HIIE increases glucose tolerance and insulin sensitivity (Cockcroft et al., 2015). This response to HIIE is due to a closed integration between the different body systems, especially the metabolic and immune systems. Recently, BDNF, more known for its neuro-regenerative function, has also been related to metabolism, with energy challenges leading to increased concentrations (Marosi and Mattson, 2014). In the present study, both protocols led to energy stress, demonstrated by lactate which increased in both HIIE1.25 and HIIE2.5 immediately after exercise if standardized to basal values justifying, at least, the BDNF behavior immediately and 60 min after exercise (Table 2). These results are according to studies by Griffin et al. (2011) and Saucedo Marquez et al. (2015). However, there was no difference between HIIE1.25 and HIIE2.5 in BDNF response, even though the duration of HIIE was set differently in both protocols. Ferris et al. (2007) demonstrated that BDNF increase was higher with higher exercise intensity, independent of the duration of exercise execution (HIIE1.25: 10.8 ± 0.81 min vs. HIIE2.5: 21.15 ± 1.62 min). In fact, HIIE has a higher “stressing nature” than continuous aerobic exercise because of its anaerobic components which are suggested to lead to cerebral hypoxia that can be a trigger to augment BDNF release by the brain itself (Rasmussen et al., 2009) as BDNF is related to cell survival and neuro-protection. On the other hand, BDNF levels decreased after 60 min, possibly because BDNF was taken up by muscle tissue to support lipid metabolism (Rasmussen et al., 2009).

Regarding metabolic response, lactate has been proposed as an important glycolytically produced metabolite that is most likely released because of increased or accelerated anaerobic glycolysis and stress response (Garcia-Alvarez et al., 2014). All participants demonstrated an increase in lactate concentration during the exercise session in both conditions. When the rate of glucose metabolism exceeds the oxidative capacity of the mitochondria (Dienel, 2013), lactate assists as a critical buffer, allowing glycolysis to rapidly produce significant amounts of energy. The higher variation in glucose after the HIIE1.25 compared to the HIIE2.5 may indicate that shorter HIIE stimulated glyconeogenesis (through an increase in catecholamines); however, as the exercise was very short, this glucose was probably not used during the exercise (Lira et al., 2015). On the other hand, the clearance of lactate during the recovery period was not measured, limiting a comparison between lactate levels after both HIIEs.

Recently, we verified that 5 km HIIE augments the anti-inflammatory status (Cabral-Santos et al., 2015). However, no study has verified whether even lower HIIE duration elicits the same anti-inflammatory response. This result could be important for training prescription, especially since the rationale for HIIE is its time-efficiency.

Studies generally adopt different HIIE protocols to evaluate anti-inflammatory effects. Lira et al. (2015) submitted athletes to 4 sessions of the Wingate test at 100% of VO_2peak_ and observed an increase in IL-10 levels, inducing beneficial alterations in the resting inflammatory profile. Dorneles et al. (2016) demonstrated that 10 × 60 s at 85–90% of maximal aerobic power, separated by 75-s at 50% of maximal aerobic power (which gives a mean workload of 65.6–67.8% of P_max_), was able to induce a progressive elevation of IL-6 and IL-10 levels immediately and 30-min post exercise in both lean and overweight-obese subjects. These results suggest an important implication of this exercise in the control of chronic low-grade inflammation in obesity. Furthermore, Wadley et al. (2016) compared untrained males who undertook three exercise bouts: HIIE (10 × 1-min at 90% VO_2max_) and two energy-matched steady-state cycling bouts at a moderate (60% VO_2max_, 27 min) and high (80% VO_2max_, 20 min) intensity, and showed that IL-6 and IL-10 increased after 30 min in both HIIE and high intensity steady-state cycling, demonstrating similar inflammatory and oxidative stress responses. Taken together, these studies showed that the acute anti-inflammatory response in HIIE seems to be intensity-dependent (Cabral-Santos et al., 2015; Dorneles et al., 2016). However, our results contradict, at the least, this perspective showing that also the duration of an exercise bout may be an important variable.

As can be seen in Table 3, the delta (measure of difference in final concentrations compared with the basal; Δ) in IL-6 changes from rest to immediately after exercise was higher for HIIE2.5 than HIIE1.25, demonstrating that IL-6 level increases may be dependent on exercise duation. Additionally, IL-10 was shown to be related to the volume of HIIE, as this variable tended to be higher in HIIE2.5 than HIIE1.25 (*p* < 0.059) between rest and immediately after exercise, although at immediately and 60 min post exercise a negative delta to HIIE2.5 and a positive delta to HIIE1.25 was found showing that IL-10 was still increasing (*p* < 0.012).

IL-6 is an important mediator involved in the regulation of the acute-phase response to injury and infection (Heinrich et al., 2003). In muscle cells, contraction leads to the activation of the mitogen-activated protein kinase (MAPK) and Janus kinase (JAK)-signal transducers and activators of transcription (STAT) cascade phosphorylation (Wunderlich et al., 2013), ending with IL-6 transcription in skeletal muscle. It is important to note that the IL-6 produced and secreted by skeletal muscle has beneficial effects on metabolism, since this response exerts effects on GLUT4 translocation in the muscle that increases glycogen synthesis, insulin sensitivity in central, and peripheral organs and enhances lipid oxidation in the skeletal muscle (Al-Khalili et al., 2006; Pal et al., 2014; Cron et al., 2016), in order to provide fuel to supply the skeletal muscle during activity.

Nieman et al. (2015) showed that prolonged and intense running on a treadmill at 70% VO_2max_ until exhaustion increased muscle mRNA expression, muscle protein content, and plasma levels for IL-6, IL-8, and MCP-1, and in addition post-run muscle glycogen concentrations were negatively correlated with changes in muscle IL-6 protein content. Concurring with these data, the present study demonstrated a volume dependent increase in IL-6 levels.

Additionally, IL-6 modulates anti-inflammatory processes, as this cytokine is able to increase the production of anti-inflammatory cytokines such as IL-10 and IL-1ra (Petersen and Pedersen, 2005). IL-10 is important in the anti-inflammatory response and promotes the preservation of IkB, thereby causing inhibition of nuclear transcription factor kappa B (NF-kB), the main transcription factor of TNF-α (Petersen and Pedersen, 2005)—a cytokine with a pro-inflammatory character that acts on the regulation of insulin sensitivity and also induces lipolysis. Recently, Cabral-Santos et al. (2015) showed markedly increased IL-6 and IL-10 levels after HIIE (1:1-min at vVO_2max_, 5 km run) in trained subjects, suggesting an important role of HIIE in blocking a possible persistent inflammatory milieu.

Our data did not show changes in the concentrations of MCP-1. Further studies are needed to better understand the mechanisms involved and the effects of different exercise modalities, intensities and volumes.

Beside the effects of different HIIE volumes on anti-inflammatory status, highlighting its potential as a feasible form of exercise prescription for coaches who want to avoid overtraining or overreaching status, low volume HIIE can be performed, since no modifications in inflammatory parameters were found after the low volume HIIE in our study. Nevertheless, other studies are warranted to verify the chronic effects of low volume HIIE on other parameters to confirm its utility. However, as shown by Wallner et al. (2013) even high intensity intervals at vVO_2max_ may lead to rather low lactate responses in case the overall mean load is low and the duration of the strenuous interval bouts is short showing the need for further studies systematically varying the variables of interval exercise.

In conclusion, this study provides preliminary evidence to suggest that HIIE may be an effective intervention strategy for promoting elevations in BDNF concentrations and in the inflammatory response of IL-6, thus both protocols were rated equally. Furthermore, the volume of HIIE seems to influence the time-response of IL-10 levels of plasma, once in the lowest volume the response of IL-10 was sustained. Both protocols are suggested to lead to improvements in the anti-inflammatory status although the maximal/optimal number of repetitions (duration) as well as the impact of them mean exercise intensity is not clear at the moment.

## Author contributions

Substantial contributions to the conception or design of the work—CCS, CIMC, and RM. Analysis and interpretation of data for the work—PM and FL. Drafting the work or revising it critically for important intellectual content—DI, PH. Final approval of the version to be published—FL. All authors agree to be accountable for the content of the work.

## Funding

This work was supported by the Fundação de Amparo à Pesquisa do Estado de São Paulo (FAPESP, Brazil) under Grant n° 2013/25310-2.

### Conflict of interest statement

The authors declare that the research was conducted in the absence of any commercial or financial relationships that could be construed as a potential conflict of interest.
