# Corrigendum: Inflammatory Cytokines and BDNF Response to High-Intensity Intermittent Exercise: Effect the Exercise Volume

**DOI:** 10.3389/fphys.2016.00662

**Published:** 2017-01-04

**Authors:** Carolina Cabral-Santos, Carlos I. M. Castrillón, Rodolfo A. T. Miranda, Paula A. Monteiro, Daniela S. Inoue, Eduardo Z. Campos, Peter Hofmann, Fábio S. Lira

**Affiliations:** ^1^Exercise and Immunometabolism Research Group, Departamento de Educação Física, Universidade Estadual Paulista – Presidente PrudenteSão Paulo, Brazil; ^2^Laboratório de Fisioterapia Desportiva, Faculdade de Ciências e Tecnologia, Departamento de Fisioterapia, Universidade Estadual Paulista – Presidente PrudenteSão Paulo, Brazil; ^3^Department of Physical Education, Federal University of PernambucoRecife, Brazil; ^4^Exercise Physiology, Training and Training Therapy Research Group, Institute of Sport Science, University of GrazGraz, Austria

**Keywords:** high-intensity intermittent exercise, inflammatory response, metabolic response, cytokines, low-grade Inflammation

In the “Abstract” Section, there was a mistake in the BDNF-values of the HIIE1.25, once the values of the standard deviation were spliced and did not contain the comma. HIIE1.25: 9.71 ± 306 is incorrect, the correct value is HIIE1.25: 9.71 ± 3.06.

In the last sentence of the “Abstract”, the word “duration” was misspelled as “durantion”.

In the “Results” Section, the last word of the first paragraph there is an error in spelling the word **duration**, appears as duation.

Finally, in the “Discussion” Section, the last sentence of the fourth paragraph has been modified from: “However, after 60-min, the IL-10 levels had returned to baseline values in both protocols.” to “However, after 60-min, the IL-10 levels had returned to baseline values **only in HIIE1.25 km protocol**.”

The authors apologize for these errors. These do not change the scientific conclusions of the article in any way.

## Conflict of interest statement

The authors declare that the research was conducted in the absence of any commercial or financial relationships that could be construed as a potential conflict of interest.

